# The new narrative medicine: ethical implications of artificial intelligence on healthcare narratives

**DOI:** 10.1007/s40592-025-00256-z

**Published:** 2025-06-08

**Authors:** David Schwartz, Elizabeth Lanphier

**Affiliations:** 1 University of Central Florida, Orlando, USA; 2 Cincinnati Children’s Hospital Medical Center, Cincinnati, USA; 3 University of Cincinnati College of Medicine, Cincinnati, USA

## Abstract

While on the surface the rapid adoption of generative artificial intelligence (AI) in healthcare signals a new technological innovation that may sideline the social sciences, arts, and literature comprising the medical humanities, we argue that one way to understand the applications of AI based on large language models (LLM) in healthcare is as a deeply narrative project. LLM-based AI endorses narrative and humanistic value insofar as it is trained on vast amounts of narrative data as inputs and generates outputs in narrative formats. We contend that the medical humanities, rather than being replaced by AI in healthcare, are all the more essential to understand the practical and ethical opportunities and constraints for responsible use and integration of such tools. By analyzing two case studies of generative AI in healthcare reported in literature, we show that narrative medicine and the medical humanities provide crucial theoretical frameworks and disciplinary skills to assess, integrate, and critique the roles and impacts of such technologies in healthcare. Rather than offsetting narrative practice via AI, responsible use of LLM-based AI in healthcare requires attention to the functions of narrative in medicine and the importance of the medical humanities.

## Introduction

Generative artificial intelligence (AI) and machine learning (ML) technology offer a range of potential benefits to healthcare systems; their anticipated value is in part alluded to by increasing business investment, rising media coverage, and rapid institutional adoption. These tools—which we’ll refer to in aggregate as generative AI —showcase opportunities to remove “guesswork from diagnostic and treatment strategies[,] … enhance medical imaging … [to assist] in early identification and diagnosis of illnesses,” and make healthcare information more accessible (Nayak 2023, p. 18). We are specifically focused on generative AI based on large language models (LLM), which work by way of a kind of capacious language liaised to humans from a binary dialect. For the purposes of this article, we cover less how these systems interact with narrative inputs and outputs and instead explore the ways in which AI’s integration contributes to and affects human interaction with and conception of narrative in healthcare.

The unfettered expansion of generative AI risks causing its integration into hospital systems to be unchecked and therefore compromising, given a host of concerns about LLM-based AI including: security breaches and information vulnerability; the generation of erroneous or inaccurate claims; reproductions of bias and stereotype; climate impact and resource demands; negative impact on critical thinking skills; and, specific to the healthcare context, the “erosion of clinical competence” (Moodley 2024). These are in addition to ethical critiques that generative AI is trained on materials without permission, plagiarizes, and creates homogenous written outputs regarding both format and content. Amidst these concerns, AI innovation and adoption continue in healthcare as across society, sometimes eagerly and other times reluctantly, with its burdens and costs generally underacknowledged.

Enter the medical humanities, a burgeoning, multidisciplinary field whose innovations, benefits, and costs may also be, like AI, variously theoretical, uncertain, overly hyped, or underacknowledged. As a field, the medical humanities reflect the “narrative turn” popularized in medicine and bioethics at the end of the twentieth century. The medical humanities are not synonymous with, but can encompass, approaches like narrative medicine, which is popularly characterized as “medicine practiced with the narrative competence to recognize, absorb, interpret, and be moved by the stories of illness” (Charon 2006, p. vii), although it can include practices beyond the direct provision of clinical care. Both narrative medicine and the medical humanities incorporate the arts, humanities, and social sciences into the giving and receiving of humanistic healthcare and to the very formulations of “health” and “healthcare.” While the medical humanities have been known to directly contribute to academic medicine and health sciences by improving empathy, perspective, cultural competence, relationship-building, resilience, satisfaction, confidence, pedagogical skill, institutional impact, ethical inquiry, and burnout detection (Remein 2020, 7–8), they too have been challenged in proving value along the same quantitative, outcome-based measures constituting an “evidence-based” threshold in medicine and demarcating efficacious science under capitalism. Quite simply: The practices and perspectives of the medical humanities currently require additional time, energy, and investment that resist easy quantification to demonstrate value.

However, instead of being relegated to a buzzword, “pipeline” for medical trainees, or “offering” to complement other trainings, narrative medicine and the medical humanities provide crucial ways to assess the roles and impacts of innovative technologies like LLM-based AI in healthcare—as well as offer fundamental frameworks for contextualizing these forms of AI, their limitations, and their potential to contribute to concurrent inquiries about ethical use and uptake. In fact, there needs to be a larger narrative *re*-turn that accompanies what appears to be inevitable shifts toward LLM-based technology as a tool not only of healthcare treatment and advancement, but healthcare interaction.

This narrative re-turn can be achieved through (a) medical humanities scholars orienting toward LLM-based AI tools to contribute more effectively to analyses, education, and evaluation of such technology in healthcare; (b) institutions that deploy LLM-based AI investing in medical humanities scholars and tools to inform policies and practices; and (c) healthcare workers gaining awareness of how their jobs, thought processes, and livelihoods are affected by LLM-based AI’s newfound implications and effects on narrative construction in their work.

## Narrative data

We find it particularly intriguing that, while the processing mechanisms of generative AI and deep learning are comprised of algorithmic predictive value, the inputs and outputs  of these systems are largely narrative. Certainly, LLM-based AI can be trained on and generate any number of things, including images and sounds. However, our focus is on large language models in which computer systems scour data found within and amongst texts, often for narrative components, including characters, information, descriptions, events, chronology, language, genre, and form, to build patterns and generate a predictive text-based output. Humans often prompt these tools by way of narrative: asking a text-based question—as if having a kind of conversation—and then consume subsequent responses/outputs as textual narratives they then integrate into their extant understanding. Human prompting sometimes explicitly requests generative AI/ML tools to compose outputs “in the style of” a particular writer, form, or rhetoric, or to “take the role of” a doctor or therapist. And many popular LLM-based AI platforms disseminate outputs via text rife with aspects we commonly associate with narrative—contextual positioning, hierarchical organization, theme-based segmentation, linked citations and references (regardless if citations are accurate or exist), mimicking a particular narrative format. These recognizable, artificially synthesized narrative-based outputs thereby in some ways perform legitimacy and compatibility for human integration, parroting reasoning, styles, and sources a human agent would produce. Ultimately LLM-generated outputs can shape an agent’s narrative conceptualization, creating expectations for LLM-generated outputs, but also for narrative products and forms in general. This not only shapes how generative AI and humans construct narrative, but reinforces and affirms (certain forms of) the genre of the narrative output as a functional source of input. However, largely absent from the integration strategy of LLM-based AI in healthcare is the perspective of narratologists and narratological ethicists.

“Narrative” resists any singular definition, in part because “[n]arrative imagining—story—is the fundamental instrument of thought. … It is a literary capacity indispensable to human cognition generally” (Turner qtd in Richardson 2000, p. 168). Richardson nonetheless collocates four main definitions of narrative, which include representation of events and/or time, presentation of causal connection(s), statements of/about events, and a reading *of* text in and of itself (2000, p. 169).

Another way to understand narrative considering its “definitional and functional pluralism” is to break down its conceptualization into premise and practice (Lanphier 2021, p. 211). The former are the components that comprise our human comprehension, such as “attributions of subjectivity/objectivity…; narratology, rhetoric and discursive analysis; storytelling structures; and narrative evidence.” Premises, on the other hand, are the processes, methods, and tools by which narrative is engaged.

Despite this pluralism, one can find productive insight about narrative by underscoring the frequency with which the word “representation” crops up in its definitions, as well as the usage of narratives themselves—in some ways comprising purpose, utility, form, and/or impact (Rudrum 2005). (Rudrum goes on to argue “that such classifications as ‘narrative’ and ‘non-narrative’ are at best provisional, inconsistent, and non-mutually exclusive” [200].) While narrative defies simple denotation, it is undeniable that narratives in healthcare are composed of representations (remarks/photos/labs *of* the body vs. the “actual” body, say) with specific usage (to record, communicate, understand, diagnose, heal, etc.).

But the purpose of this article is to investigate the integration of narrative-focused generative AI into healthcare, which, as an industry, primarily sees narrative practice as complementary or supplementary rather than core. As explained, the corpora crucial to LLM-based AI are troves and troves of narratives that are processed as training data: nonfiction and fiction, first-hand accounts and scholarly research, outdated studies and conspiracy theories, translations (both accurate and inaccurate), copywritten and public record and bot-generated reiterations, and so much content generated foremost for advertising and marketing, all modulated in part by digital cultural representation and participation, which then is gathered and synthesized and scraped. This is, as many have pointed out, cause for concern, given these sources contribute data points regardless of their quality or validity and at times disassociated from their context, authorship, and reception (Weise and Metz 2023) all while amplifying certain biases (MIT). AI therefore reflects a skewed version of life, the way the world is not exactly, and it does so through its consumption and production of narrative.

There’s been much handwringing about the state of humanities education in general and perhaps a sense of foreboding that, despite significant medical humanities uptake, this new era of AI could further distance medicine from the humanistic, if not the humanities (Madsbjerg 2017). Yet we live in a time entrenched in narrative by way of the internet and technology trained on it: medicine backed by LLM is a kind of new narrative medicine, for which the recent decades of narrative attention in medicine, the careful scrutiny of how and when and why of what we do with narrative, is exactly the attention and skills necessary for the new narrative machine.

The quantum impact of the narrative turn in computing is echoed in a similar turn emerging out of a rapid technological advancement and automation in healthcare that some have argued depersonalize medicine (Grebenshchikova 2023). And so narrative attention is now postured as a counterpoint to the modern acceleration of healthcare which was moved away from a humanistic core for the sake of cost-effectiveness (Moodley 2024). The original narrative turn was no single approach or insight, but rather a collection of theories, philosophies, and practices. Perhaps most famously—though far from exclusively—Rita Charon’s concept of narrative medicine that aims to arm healthcare workers with alternative competencies to improve healthcare is one component of a narrative turn that was well underway by the time her theory emerged (Lindemann 1997).

This set up an important counterbalance to purely efficiency-based healthcare. Narrative methods in medicine are generally conceived of as alternatives, or antidotes, to increasingly impersonal and technological healthcare delivery (Murphy 2015). For example, largely in response to the emergence of electronic medical records (EMR), Charon proposes engaging in a “parallel chart” practice by which providers maintain narrative documentation, possibly co-constructed with the patient to enable them to weigh in and/or correct this record (2006). This strategy counters flaws from EMR that shape the nature and form of patient recordkeeping, in part informed by its rollout. While EMR was developed in the 1970s, it wasn’t until the American Recovery and Reinvestment Act of 2009 that hefty implementation cost could be overcome for mass adoption (Honavar 2020).

EMR was designed for the efficiency of capturing, recalling, and sharing information, as well as integrating into partner providers and systems, including billing systems. Charon’s narrative project of the “parallel chart” thus proposes a record system that would *add* inefficiencies in some senses—it literally takes more time to complete. However, it increases other kinds of efficiencies by in theory providing more compassionate, humanistic, and higher-quality care that may be better able to identify healthcare problems or needs and partner with a patient to address them effectively. It’s important to note that in practice EMR does not necessarily support optimized efficiency as evidenced by the interest in AI charting to offset provider documentation burdens and permit providers to return to more face-to-face time in clinical encounters (Dahdah 2024)—in other words, to offload electronic burdens to also enhance humanistic, compassionate care. At the same time, generative AI technology is poised to offload and approximate humanistic skillsets into a computer-automated workflow that allows providers under pressure from hospital administrators and insurance companies to further “streamline” their approaches to intervention and increase their rate of care.

Narrative is operating constantly around us whether we attend to it or not. It not only reflects our world—or the pieces we choose to narrate of it—but it also shapes our perception of that world, and therefore our understanding of it. LLM-based and generative AI has the opportunity to reify this reality through its capacity to consider and compare the individual in relation to statistics-based medicine. In doing so, it might situate the individual accurately, but it also can lose the individual to a larger—perhaps inappropriately applied—narrative construction with which a patient or their provider may not concord. This is a known issue in medical risk predictors by way of their function, but with AI’s scale and integration, this problem might be exacerbated such that certain individuated narratives become so obfuscated through aggregate, statistical, predictive pattern imposition that non-conformist datapoints are systematically irradicated. The data, in this scenario, are people.

This question of personal experience versus statistical representation has been a theme of the medical humanities by way of critical readings of texts such as Charlotte Perkins Gilman’s “The Yellow Wallpaper” and the literary theory espoused in works like Susan Sontag’s *Illness as Metaphor*. Providers will recall the Nightingale Pledge, the Hippocratic Oath, and patient diagnostic interviews. The sick shape their stories from hospital beds, their supporters will tell them new ones, and providers will incorporate this in what they know scientifically to be true.

In fact, there is no medicine unbounded from narrative. The prioritization of efficiency in privatized healthcare, the overwhelm of additional tasks of healthcare staff,^1^ and the alluring innovations of new technologies have demarcated a false dichotomy between evidence-based medicine and narrative medicine (Misak 2010). The former is considered optimized, integrated, scientific, and rational; the latter can be chided as alternative, associative, humanist, and creative. But just as there is no medicine without humans, there is no humanity without narrative. Now, in the AI revolution of medicine, the false distinction between narrative and evidence further collapses. When it comes to LLM-based AI, narratives are data, and data yield narratives. The question is, in part, whether we can recognize and reconcile them as interconnected, or if the AI turn creates the spurious sense of living amidst “data-driven” evidence that is immune to narrative subjectivity.

And so, we might therefore demarcate—not define—new narrative medicine as a necessary evolutionary turn in the medical humanities whereby our conceptions of and engagements with narrative in health and healthcare accommodate that which is produced, consumed, and exchanged through ongoing technohumanist collaboration.

## Ethical narratives in healthcare

While LLM-based AI poses as a technological pivot to minimize certain human errors and relieve certain burdens in healthcare (Moodley 2024), its inextricability from narrative input (the very data from which large language models draw) and narrative output (reproduction of extant genres in medicine like chart noting or patient communications) fundamentally shifts the role of the healthcare worker vis-à-vis narrative production. As we’ll explore in our case studies, one promising change might be that healthcare workers are freed from other tasks to allow for more interactive time with their patients. But this too affects the very nature of how healthcare workers—and possibly their patients—then interact with the narrative content of those encounters. Responding to this sea change requires practices that establish, monitor, and enforce an ethical narratological system to guide generative LLM-based AI in healthcare, as well as the ways those outputs affect both workers and patients. Even though healthcare providers remain more agential in the co-production, shaping, documentation, and interpretation of narratives in comparison to their patients, generative LLM-based AI technology in clinical documentation shifts clinicians’ role from authors to editors, a compromise for which medical training and hospital systems will need to account.

Rather than ceding the floor exclusively to technology and technological expertise, to accommodate this narrative turn, hospitals and healthcare groups should invest in the medical humanities. Ethical AI integration requires narrative awareness and practice, both of which are in the service of ethical medical practice. To better understand the implications of this, we’ll turn to two case studies.

### “More correct than incorrect”: ChatGPT in the PICU

If implemented accurately and efficiently, the potential value of computer-guided information gathering and assessment includes providing patients and data systems with precise and trustworthy insights, which could result in “high satisfaction, less stress, and improved participation in shared decision-making” (Hunter et al. 2024, p. 2). But what is lost by digitally offsetting information gathering and assessment, and how will hospitals and care networks accommodate these newfound tools?

A recent study researched the effectiveness of ChatGPT (a generative LLM-based AI tool) in employing patient-specific information “to provide high-quality answers to parental questions in PICU clinical scenarios” (Hunter et al. 2024, p. 1). The study evaluates ChatGPT responses based on completeness and accuracy, but also empathy and understandability (Ibid, p. 3), which invites consideration of a healthcare worker’s role once previous humanist engagement is successfully offset to computers, and, in the case of empathy, expected from them. While “several authors have noted human-like reasoning as an emergent capability of LLMs,” (Ibid, p. 7) our focus is instead on the fact that while technological solutions may address some key challenges of healthcare (documentation, labor, time, efficiency), we need to be cleareyed about how, by way of their potentially outsized impact, such solutions require *greater* attention to medical humanities, especially understanding of narrative practice and ethics. Enter ChatGPT, which has what seems like infinite resources and attention. But it doesn’t: While AI suggests the possibility of improved efficiencies in the future, its current negative environmental impact is “less clear and often underestimated” (Coulson 2024), and its harms—to the planet, critical reasoning, etc.—need evaluation. AI already contributed to “2% of the world’s overall carbon footprint,” and “this number is expected to rise if the industry fails to curtail its energy consumption” (Mehta 2024).

Empathy is a popular subject in the medical humanities, particularly in teaching or honing the empathy expected from healthcare communications, which are increasingly being offloaded to machines that some find “more compassionate than expert humans” (Ovsyannikova et al. 2025). Can machines be empathetic, or do they approximate a bare minimum of what patients need of empathy, not dissimilar to how other industries have skimped on composite offerings in the mid- and post-pandemic wake of “shrinkflation”? Morrow et al. report that “AI technologies are being used to enhance empathetic awareness, empathetic response and rational behavior; communication skills; health coaching; therapeutic interventions; moral development learning; clinical knowledge and clinical assessment; healthcare quality assessment; therapeutic bond and therapeutic alliance; and to provide health information and advice” (2023, p. 2). In fact, the patient-preferred portrayals of empathy might be actualized in the *customization* of communications that generative AI provides (Zohny et al. 2025). Of course, a program trained to provide responses perceived as “empathetic” is—or ought to be—different from actual empathic interactions, raising questions as to whether empathy in the technoscape of late-stage capitalism is in fact indiscernible from the performance of and suggested commitment to empathy. Without properly understanding technology’s relationship with and impact on empathy, healthcare institutions run the risk of transmuting empathetic interactions into instances of price, labor, and time—not dissimilar to paying more to choose your seat on an airline—rather than the transformative, humanistic interactions which defy limited capitalist conceptions of cost-efficiency.

The prompts used in the ChatGPT study in the Pediatric Intensive Care Unit (PICU) included data from “typical” patient assessments and treatment plans as well as simulated parental questions. The answers produced by ChatGPT were then evaluated by physicians. The doctors found the AI-generated results “had high accuracy …, empathy …, completeness …, and understandability … and no response was deemed likely to cause harm” (Hunter 2024, 1). “[I]n the study, < 3% of physician reviews rated [AI] answers as more incorrect than correct” (Hunter 2024, 7). ChatGPT was right more than it wasn’t. Such a tool could, in theory, reliably take pressure off doctors and nurses to handle empathic information communication. However, the study failed to test for parental evaluation of empathy and understandability. It will be important for institutions to study the differences of empathetic perception and expectation amongst providers, caretakers, and patients. And the incorporation of additional actors poses a question about the ways in which patients, doctors other hospital staff, support systems, and computers must all be looped in to inform one another. Each will excel in and participate toward different kinds of narrative production.

Moreover, depending on how such a tool is used, it could also distance participants by centralizing communication flows through LLM-based AI rather than the patient’s human healthcare team. While this might help parents receive accurate and understandable answers to their medical questions, it might also diminish the medical team’s awareness of parental concerns thus eroding rapport or the ability for the team to anticipate problems or engage in a shared decision-making process, and it might lessen the medical team’s sense of parental understanding of their child’s diagnosis or treatment, which is important for informed consent.

On the one hand, one could imagine how this might compromise a collaborative narrative about the patient’s care or condition. Instead, aspects of clinical understanding and questions would get distributed with a ChatGPT intermediary as in a game of telephone, potentially inhibiting comprehension and thereby subsequent empathy and action. On the other hand, some of this may already occur amongst humans, when healthcare workers come on and off service within teams, passing off information between each other or between patients and their families. And as we’ve seen with telemedicine, technologies that may appear to keep patients and healthcare workers at a distance can also be a means to greater connection and intimacy. For the patient who may not otherwise present in person for care, telemedicine can be an effective way to triage needs and increase accessibility of care. . Making sense of these different potential frameworks for understanding how technologies shape clinical encounters, and identifying and evaluating the tradeoffs they pose, requires integrating medical humanities scholars into the health systems utilizing these tools, and into the project of a new narrative medicine.

This case study illustrates a clear opportunity for how “ChatGPT could generate patient-specific responses by using unprocessed text from medical assessments and plans that include real-world elements like acronyms, bullet points, and incomplete sentences” (Hunter 2024, p. 7). In other words, it can quickly summarize a significant amount of information in forms generally inaccessible and nonsensical to a layperson with access to a patient’s medical record, with what appears to be reasonable reliability, yielding a new kind of narrative production for healthcare. (A study comparing the ability of ChatGPT to a human medical professional to produce understandable, accurate, and empathic responses to the same prompts would enhance an analysis of the potential benefits and constraints of ChatGPT as an information source). But one way to understand the narrative function of such LLM-based tools, then, is not as generators or arbiters of patient narratives, but as summarizers, collectors, and anthologizers. This preserves nuanced authority for each human actor within the care process but also allows tools like generative AI to offset labor and to fill in the gaps, highlight what *might* be most important, and connect threads together. To ensure ethical consolidation, a role or task will need to emerge to monitor and evaluate AI’s contributions, particularly as these tools still remain nascent.

One of LLM-based AI’s current use cases is the ability to offload and streamline mundane tasks, such as generating email exchanges and cover letters, which are then, depending on corporate resources, evaluated by AI yet again. There is a similarity here to how generative AI is filling in for classroom study guides, or websites like SparkNotes and Wikipedia. While these tools can give remarkably informative summaries about the timelines, plots, and core themes of a text, they cannot say much that is overtly unique, specific, and interesting without user-guided parameters. En masse their responses are by the books generalized overviews—not dissimilar to encyclopedias, though currently less detailed and often less interesting; this anodyne style is hallmark to the AI/ML voice, aiding in their identification (Chiang 2024). In this sense, generative AI has its own genre that reads as, well, AI-generated.

LLM-based AI in hospitals then can easily handle the summation and generalization of narratives, particularly in already established genres (like the chart note, or the electronic portal patient reply), which enables healthcare workers to weigh-in with their expertise by way of detailed counter-, extra-, or meta-narrative. Similarly, AI can work to consolidate patient outputs into streamlined stories to perhaps expedite or improve diagnostic approach. Often in health humanities, the idea of “co-creation” is the establishment of a health or illness narrative *between* a clinician and patient. Yet in this two-way model of general vs. specific, technological vs. human, between humans and LLM-based AI, there is a potential that: (a) workers, patients, and support communities co-create narratives, based on the most first-hand results of care, personal experience, human connection, and borne witness; (b) autotextual, AI-generated narratives summarize and compare the patients’ realities and provider recommendations to average, statistical experiences; and (c) subsequent data, studies, and results augment and inform both (a) and (b). But notice, this multistep model is rather narrative *intensive.* It does not offload work overall for healthcare workers as much as it changes the nature of the work, and at best, enhances the results of the work by requiring new narrative skills and processes from humans and AI.

The role of generative LLM-based AI in healthcare is therefore not dissimilar to existing statistical tools in medicine, such as the Kaplan–Meier estimator, which can be used to calculate patient survival rates. These charts are helpful by providing generalized context for both providers and patients; however, the challenge of such a predictive curve is in whether any particular patient will ultimately end up being a data point that  conforms or proves anomalous. This question of conformance or individuation is, as we know from stories, how heroes are established; but unlike other tools, generative AI will take the details as data and shape it to a story of its own. Paying attention to LLM-based AI’s benefits—including its ability to aggregate—and consequences—that it is prone to obscure, bias, or exclude, as well as occasionally fabricate—are essentially narrative strengths and weaknesses, requiring careful attention not only from computer engineers, but also medical humanities scholars.

### We are the existing workflow: DAX copilot

Microsoft’s DAX Copilot software listens ambiently to multiparty conversations to draft “specialty-specific standardized … clinical summaries that integrate with existing workflows” (Dahdah 2024). The company claims that the AI tool has enabled doctors to “see an average of 11.3 additional patients per month,” spend “24% less time on notes,” and decrease late-night administrative hours by 17%. What’s more is how 77% of users self-reported that the “solution had improved the quality of their documentation,” with 81% saying it reduced cognitive burden.

As cultural theorist Sianne Ngai details in *Theory of the Gimmick* and journalist Oliver Burkeman outlines in *Four Thousand Weeks*, the streamlining offered by technology can easily lead to additional work output, rather than less. With DAX, through studied increased efficiency, doctors are seeing more patients—though this does not necessarily entail longer time spent with each patient or a higher quality encounter—such data remain to be reported. Yet, for a patient seeking a medical consultation, being seen by a provider is perhaps the most crucial step for establishing a care plan in the interest of their health. And so, while technology may on the surface increase access for more patients, and minimize inefficiencies for clinicians, it may come with cost: “increased clinical effort and excess time using the electronic health record (EHR) are known contributors to physician burnout” (Saag 2019). While documentation burdens may lessen, the tradeoff may be increased clinical effort, with significant interface with the EMR remaining.

Moreover, in comparison to a clinician’s interface with a traditional EMR, this software’s ability to listen into patient encounters and write summary notes cleaves the role of the in-person visit: technology instead is responsible for (at least some of) the work of narrative attention in the clinical encounter. In a sense, this removes the first-person subjectivity of the clinician to author EMR notes. The clinician and patient are co-constructing the narrative reality of the clinical *encounter* in their discussion. The generative AI tool then summarizes the clinical encounter from a kind of omniscient narratorial perspective. It is not the clinician’s perspective on what happened in their visit with the patient; it is an allegedly neutral third-party perspective. Thus, in other ways, this software introduces a sort of third-person subjectivity through indexing the encounter against all other encounters in aggregate to make sense of it, rather than addressing the actual subjectivity of the particular parties involved. This raises questions as to how the LLM-based AI is trained.

We might be inclined to view DAX Copilot as an omniscient narrator. But it isn’t *all-*knowing; its “perspective” is an amalgam of the data on which it is trained and not the data it is missing. This is not dissimilar, maybe, to the omniscient narrator in a work of fiction that appears to the reader as all-knowing from within the text, but of course is built by the notably non-omniscient author and shaped by the aesthetic and artistic opportunities of the piece and the world therein. Among the ethical risks in healthcare is the perception of neutrality where it may not exist. For example, if training data are significantly shaped by existing chart notes in EMRs, then the style and perspective of clinicians may unduly influence the documentation product from the Copilot tool, even though it appears to be a more neutral third party bearing witness to and summarizing the encounter. And the tool’s interpretation of events may unreasonably influence a patient’s record. Imagine a patient shares at a medical visit that they have not been able to take a medication due to transportation limitations to the pharmacy. Does a tool like DAX Copilot summarize this as “transportation barriers impede consistently accessing prescription refills,” thus identifying the problem as transportation,  indicating social needs the medical system could support? Or does it summarize simply that the “patient is non-compliant with medication” using stigmatizing language it may have learned from other patient records, and which does not illuminate the structural determinants of health needing to be addressed?

Microsoft claims these outputs are *draft* summaries, which require the clinician, in a newly emergent role, to edit and attest to the AI-generated notation. And this editorial role shares a family resemblance to other practices. Previously, when clinicians dictated their notes into voice-to-text software that they then edited for accuracy, they remained the author of their words with technology serving as scribe. Attending physicians also may play editor when reviewing and attesting to notes written by trainees, but the author’s name remains attached to those notes with the attending co-signing. A clinician editor of an entirely externally generated—but simultaneously experienced—narrative is, we believe, meaningfully distinct from the aforementioned paradigms, introducing new questions, particularly related to privacy (including how the LLM-based AI trains on the encounters it “listens in” on or obtains feedback from clinician-corrected summaries to improve its output) and authorial agency.

For example, according to its product page, DAX Copilot is trained on over 10 million encounters to ensure its expertise within the genre of healthcare narrative. But as more notes become AI-generated, and subsequent AIs are trained on those notes, how will this influence EMRs? They will no longer be the human documentation of medical encounters, but the technological accounts of how medical records were supposed to document medical encounters. Researchers are already finding that AI trained on AI content “collapses,” offering depreciated responses in terms of accuracy and applicability (Shumailov 2024). As we move into a mirror world of AI generation occasioning amplified degradation of medical narratives, what happens to clinician understanding and patient articulation? If we increasingly experience healthcare in a world of AI-generated health expectations and information—a simulacrum of interpersonal exchange, vulnerability, and care—do we too become as limited and prescribed as our digital arbiters? And then what will happen to our health, which is not just scientific, but is also humanistic?

Regardless of the benefits afforded by technological interventions, especially for clinician time and patient access—and we must think critically about how and why these tools are supporting healthcare workers and systems—the human agents will become more and more removed from the narratological production of clinical encounters. There is a price for this. While humans will continue to co-produce these meetings and edit their records, the summation, articulation, and narrative structuring will be the responsibility of generative AI. By offsetting narrative efforts, healthcare workers may not just outsource a task, but in fact also outsource aspects of their professional autonomy and the self-determination of their patients. While LLM-based AI showcases important compromises regarding information access and potential bias with healthcare narratives, by repositioning these roles, we reposition the agential hierarchy with humans ceding to technology. At the scale and speed with which AI functions, we give over the reins and allow it to in part define what healthcare is for us—and therefore our health.

## Conclusion

Ultimately, the emergence of LLM-based generative AI as a narratological production agent in healthcare shifts the role of healthcare workers from authors to editors of the documentation resulting from their interactions. The implications of this are far-reaching.

First, such generative AI may erode overall narrative awareness on the part of human agents by ceding the notion of comprehensive narration to AI/ML. This ironical phenomenon occurs by the closed, black-box system through which LLM-based AI consumes and produces information; however, the reality is generative AI succeeds at identifying patterns and surfacing what’s hidden in plain sight. Where AI/ML fails is in distributing unique narratives—and so it must attribute the rarity of one’s data to a generalized narrative about disease and/or illness. Here, narrative medicine and the medical humanities can serve not just as alarm bells but aid in better integrating computer-generated narratives into a narrative-reliant healthcare system. And perhaps a new kind of narrative awareness will emerge.

Second, LLM-based AI has the opportunity to approximate narratological components previously limited to human production. These include empathy, time and attention, awareness, and understanding. By offsetting this labor, healthcare workers could reorient toward other tasks, all while establishing new roles for those who will liaise the intersection between technology and patient as well as provider. However, these humanist traits are not mere byproducts of this technology as-is; these are features to program for, crucial components previously jettisoned to obscure catchalls like “bedside manner.” If technology is to support healthcare, it must be designed with the necessary beneficence established by way of narrative-minded providers. The imperative challenge here will be in convincing the companies producing these tools and systems to understand the value of humanistic healthcare.

Generative LLM-based AI in part reifies, at a new scale, and with greater production-efficiency, the work humans have long strived to establish. Its affiliation as technology is correct insofar as AI is code, datahouses, and processes/processors; however, its function is an intersection of both science and the humanities by way of its narrative inputs and outputs, not to mention its causes and effects. LLM-based AI in fact endorses a narrative and humanistic view insofar as it is trained on vast amounts of narrative data, all while technologizing medicine and human interaction, thus masking its reliance on humanistic and narrative input. It affords an opportunity to therefore welcome and join narrative and evidence, humanism and science, too often divided along a falsely constructed dichotomous system rather than understood as integrated. The new narrative medicine must disintegrate the erroneous binaries of humanism and science to ensure agential design of technology’s role in humanist healthcare and better evaluate value for both human and technological workers.

We hope this paper can not only highlight the heightened importance of the medical humanities as a kind of call-to-action in the advent of generative AI, but also a recommendation for how to ethically consider changing roles in narratological production. The questions medical humanities investigate, particularly about the narrative dimensions of and ethical obligations within and established by the clinical encounter, are essential to the two examples we surveyed herein. Experiences of healthcare and illness are not immune to external influence: cultural narrative shapes interactions between patients and clinicians, attitudes and conceptions of illness, and the goals and values brought as individuals and as communities to healthcare decisions. But LLM-based AI presents a new cultural force of record influence, scale, and distribution that both pushes and pulls on the clinical encounter as it becomes an efficiency tool of patient interaction and documentation. Ethical analysis of generative AI tools requires  careful attention to them as *narrative* tools.  Their embedded narrativity is essential to their ethical complexity.

The narrative role of medical professionals—who in theory may be freed up for more face-to-face interaction—will become editors rather than authors of narrative data. This may not be entirely problematic. Positioning healthcare workers as authors may in fact attribute too much agency to their narrative framing of an encounter. Inviting the healthcare professional in as editor may permit them to notice—and where necessary modify—narratives imposed on an encounter that they find inaccurate or unhelpful. In other words, it may motivate a more critical stance toward generated narratives and invite reflection on the reasoning, utility, and impact of such representation.  However, this change may also have the opposite effect: creating a kind of narrative passivity on the part of those reviewing AI-generated text and summaries that in the end merely reinforces narrative scaffolding baked into the texts on which LLM-based AI is trained, which it reproduces uncritically in new patient encounters. The latter would relegate narrative to backgrounded white noise, rather than the source of patterned understanding and human interaction. The point here is that neither of these pathways are foregone conclusions, and there is a need for ongoing medical humanities attention to these issues to test and evaluate these tools in practice. Rather than cede the floor to a technology-driven new frontier, the medical humanities should be further embedded in the inputs of these technological advances, and a source of their refinement, evaluation, and accountability.

And we encourage others to consider LLM-based AI at this present time as operating narratively as a compilation. Anthologies collect, chronicle and order narrative information, with introductions that summarize high-level themes, societal relevance, and core concepts. This puts healthcare workers into a role of being *readers*, in addition to editors and narrative co-producers. Critically reading takes more time than skimming or searching for summary, which changes how we need to consider documentation efforts and the technology that alleviates that labor. Should healthcare workers be permitted the energy and attention to read deeply, what will they find? And how will they react? Narrative medicine has long preached the value of close reading in the diagnostic setting and clinical encounter (Charon 2006), but this method for textual and narrative analysis pivotal to literary and creative writing classrooms may additionally be apt to many in healthcare and other industries given the progressively reliant interrelations with generative AI. All outputs will need to be closely evaluated for comprehension, accuracy, cohesion, logic, relevance. But close-reading too is the process through which one considers the role of the text, author, subject matter, context, and themselves as activated by the material to acquire not just understanding but something closer to meaning. It puts both objectivity and subjectivity into the reading experience so that the text—whether computer-generated, human-scribed, or patient-voiced—becomes more than just words.

## Data Availability

No datasets were generated or analysed during the current study.

## Footnotes

1 “In 2023, approximately 53% of physicians surveyed by Medscape reported feeling burned out, and 23% reported feeling depressed. Long working hours, bureaucratic demands, and loss of work-life balance due to administrative requirements are leading causes” (Dahdah 2024).
